# Monoglyceride lipase deficiency affects hepatic cholesterol metabolism and lipid-dependent gut transit in ApoE−/− mice

**DOI:** 10.18632/oncotarget.16529

**Published:** 2017-03-23

**Authors:** Nemanja Vujic, Melanie Korbelius, Christina Leopold, Madalina Duta-Mare, Silvia Rainer, Stefanie Schlager, Madeleine Goeritzer, Dagmar Kolb, Thomas O. Eichmann, Clemens Diwoky, Andreas Zimmer, Robert Zimmermann, Achim Lass, Branislav Radovic, Dagmar Kratky

**Affiliations:** ^1^ Institute of Molecular Biology and Biochemistry, Medical University of Graz, Graz, Austria; ^2^ Center for Medical Research, Institute of Cell Biology, Histology and Embryology, Medical University of Graz, Graz, Austria; ^3^ Institute of Molecular Biosciences, University of Graz, Graz, Austria; ^4^ Institute of Biomedical Engineering, Graz University of Technology, Graz, Austria; ^5^ Institute of Pharmaceutical Sciences, University of Graz, Graz, Austria; ^6^ BioTechMed, Graz, Austria; ^7^ Current address: Institute of Molecular Biosciences, University of Graz, Graz, Austria

**Keywords:** endocannabinoid, cannabinoid receptor, desensitization, lipolysis, cholesterol

## Abstract

Monoglyceride lipase (MGL) hydrolyzes monoglycerides (MGs) to glycerol and fatty acids. Among various MG species MGL also degrades 2-arachidonoylglycerol (2-AG), the most abundant endocannabinoid and potent activator of cannabinoid receptors (CBR) 1 and 2. MGL-knockout (−/−) mice exhibit pronounced 2-AG accumulation, but lack central cannabimimetic effects due to CB1R desensitization. We have previously shown that MGL affects plaque stability in apolipoprotein E (ApoE)−/− mice, an established animal model for dyslipidemia and atherosclerosis. In the current study, we investigated functional consequences of MGL deficiency on lipid and energy metabolism in ApoE/MGL double knockout (DKO) mice. MGL deficiency affected hepatic cholesterol metabolism by causing increased cholesterol elimination via the biliary pathway. Moreover, DKO mice exhibit lipid-triggered delay in gastric emptying without major effects on overall triglyceride and cholesterol absorption. The observed phenotype of DKO mice is likely not a consequence of potentiated CB1R signaling but rather dependent on the activation of alternative signaling pathways. We conclude that MGL deficiency causes complex metabolic changes including cholesterol metabolism and regulation of gut transit independent of the endocannabinoid system.

## INTRODUCTION

Monoglyceride lipase (MGL) hydrolyzes monoglyceride (MG), which derives from the degradation of phospholipids and triglycerides, to glycerol and fatty acid (FA) [1, 2]. Certain MG species are known receptor ligands and mediate signaling functions. Most prominent are 2-arachidonoylglycerol (2-AG), the most abundant endogenous ligand of cannabinoid receptors (CBRs) [3, 4], and 2-oleoylglycerol as ligand of the G-protein coupled receptor 119 (GPR119) [5]. CB1R shows both central and peripheral distribution and mediates effects associated with neuronal and metabolic regulation. Specifically, CB1R activation influences neuronal plasticity [6, 7] and pain sensations [8], reduces locomotor activity [9], gut motility [10], hepatic β-oxidation [11], and mitochondrial biogenesis [12]. It also stimulates food intake [13], body weight gain [14], adipogenesis [15–17], hepatic *de novo* lipogenesis [11, 18–20], and insulin resistance [11, 20, 21]. Overall, CB1R activation negatively affects metabolic homeostasis and thus may influence the development of the metabolic syndrome and cardiovascular diseases. CB2R, on the other hand, is almost exclusively expressed in immune cells where it attenuates the inflammatory response, reduces immune cell functionality, and causes leukocyte apoptosis [6]. Consequently, CB2R regulation of immune reactivity may have profound effects on the development of cancer, pathogen clearance, autoimmune diseases, and chronic inflammatory conditions including atherosclerosis. Therefore, MGL is at the crossroad of lipid homeostasis and complex signaling networks and is involved in the regulation of a plethora of processes associated with cognitive functions, pain sensations, inflammatory reactions, lipid and carbohydrate metabolism, and systemic energy homeostasis.

MGL knockout (−/−) mice accumulate 2-AG in the brain and suffer from central CB1R desensitization, where cell surface availability of the receptor is reduced due to its downregulation and internalization with concomitant blunting of signal transduction [22–24]. Similarly, MGL deficiency may also cause CB1R desensitization in the gut [25]. We have previously characterized MGL deficiency and the endocannabinoid system in the context of atherosclerosis development by generating apolipoprotein E/MGL double knockout (DKO) mice [26]. These mice show less vulnerable atherosclerotic plaques with increased collagen deposition [26]. Phenotypic peculiarities of apolipoprotein E knockout (ApoE−/−) mice include a hyperlipidemic plasma profile with disrupted hepatic uptake of both liver- and intestine-derived ApoB-containing lipoproteins, phenotypically resembling human hyperlipoproteinemia type III [27, 28], pronounced non-alcoholic hepatic steatosis [29], and reduced biliary cholesterol secretion but unaltered intestinal cholesterol absorption with increasing dietary cholesterol load [30]. In the current study, we used the same mouse model to explore the metabolic adaptations in MGL deficiency, which may occur as a functional consequence of aberrant lipid signaling. We found that MGL deficiency causes complex changes in cholesterol metabolism and in the regulation of gut transit.

## RESULTS

### Plasma parameters and physiological monitoring of DKO mice

**Figure 1:**
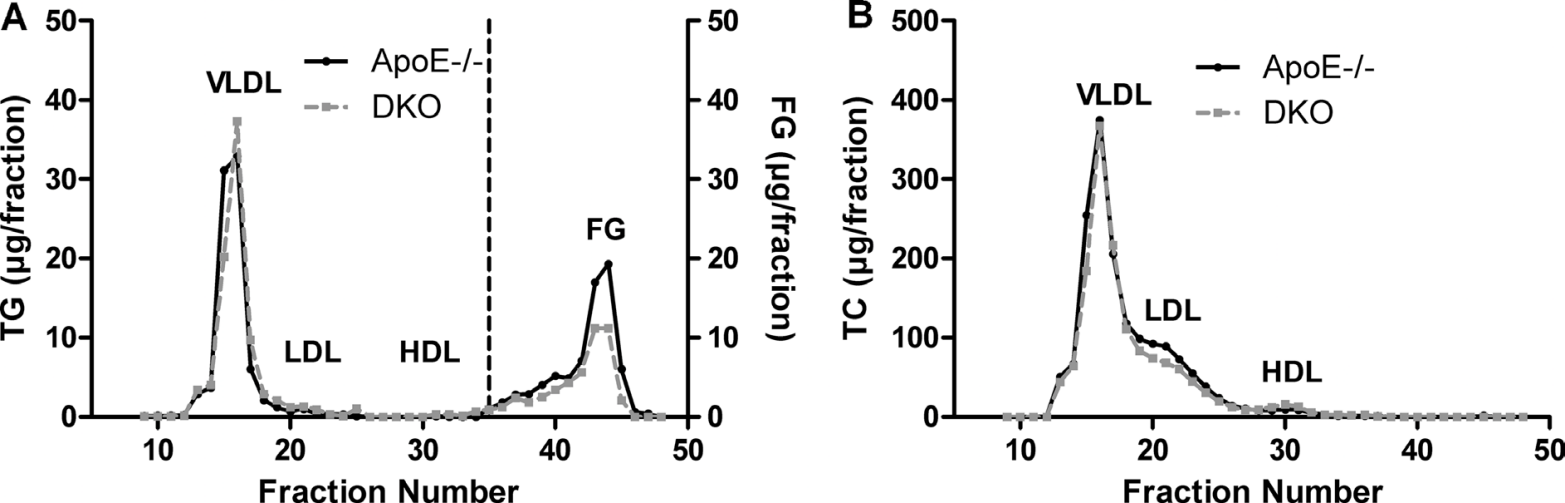
Reduced fasting FG concentrations in DKO mice FPLC analysis of plasma (**A**) TG and (**B**) TC of 12 h-fasted ApoE−/− and DKO mice fed WTD for 8 weeks. Data represent a pool of plasma samples (*n* = 5) per genotype.

**Figure 2:**
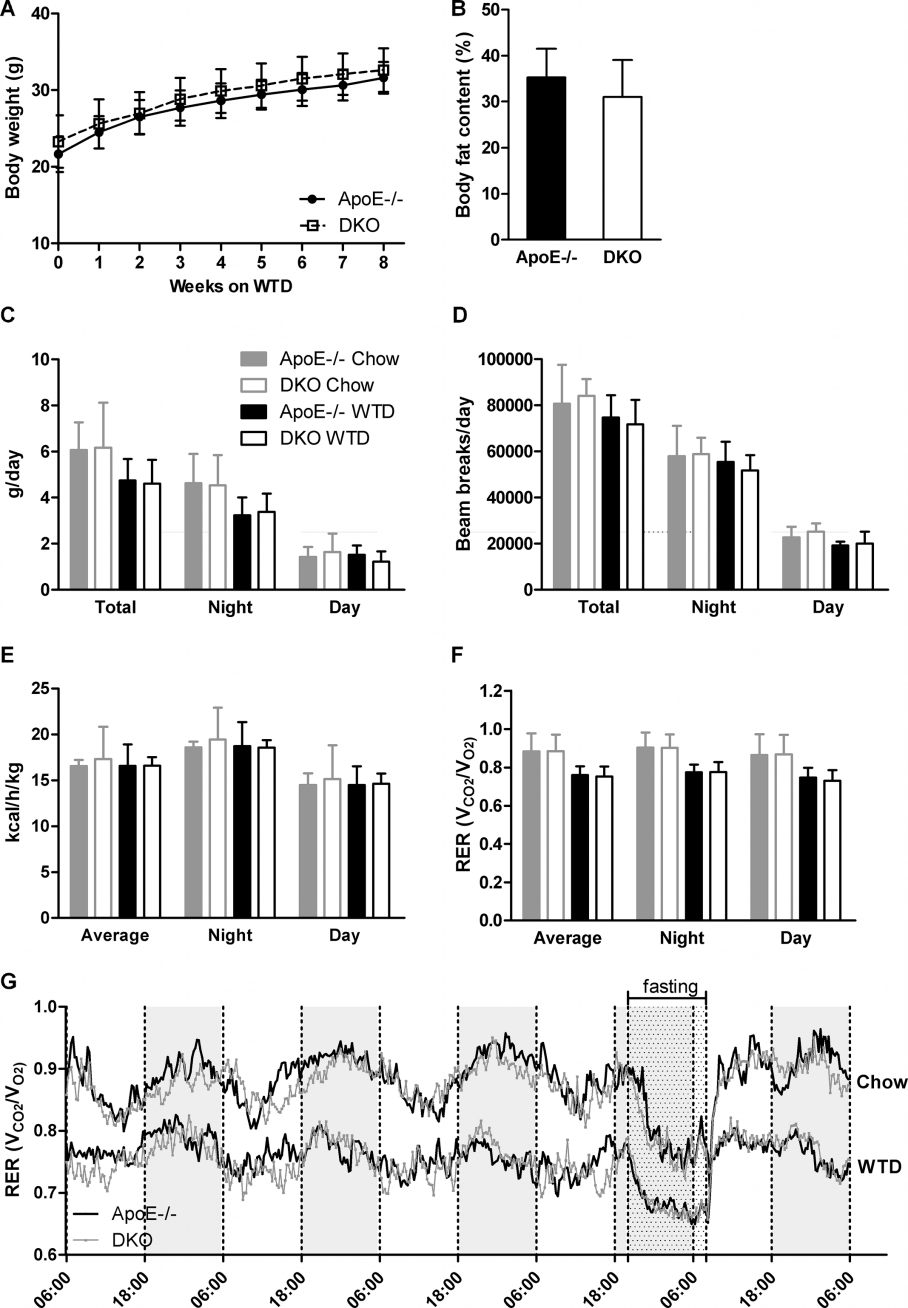
Lack of centrally mediated cannabimimetic effects in DKO mice (**A**) Body weights of ApoE−/− and DKO mice during 8 weeks of WTD (*n* = 24–34). (**B**) Quantification of relative body adiposity of ApoE−/− and DKO mice after 8 weeks on WTD by MRI (*n* = 5−6). Daily (**C**) food consumption, (**D**) locomotor activity, (**E**) energy expenditure, and (**F**, **G**) RER of ApoE−/− and DKO mice fed chow and WTD (*n* = 5). Data represent means ± SD.

Female ApoE/MGL double knockout (DKO) mice showed no significant changes in plasma triglyceride (TG) and cholesterol levels as compared to ApoE−/− mice [26]. However, a more detailed analysis of male mice revealed reduced plasma free glycerol (FG) concentrations after fasting (Tables 1 and 2, Figure 1A) with comparable TG and cholesterol lipoprotein profiles (Figure 1A, 1B). Moreover, we observed decreased plasma free fatty acid (FFA) concentrations in fasted DKO mice (Table 1), which (together with reduced FG concentrations) are indicative for reduced release of lipolytic products from white adipose tissue during fasting. The opposite effect was observed in the postprandial state in mice refed for 2 h after overnight fasting, with elevated FG and FFA concentrations in DKO compared to ApoE−/− mice (Tables 1 and 2). Body weight gain (Figure 2A) and body composition during WTD feeding, however, were unaltered between ApoE−/− and DKO mice (Figure 2B, Supplementary Figure 1). By using metabolic cages and indirect calorimetry, we determined food intake (Figure 2C), water consumption (Supplementary Figure 2), locomotor activity (Figure 2D), energy expenditure (Figure 2E), and respiratory exchange ratio (RER) (Figures 2F, 2G), which were all comparable between both genotypes. These results indicate that DKO mice lack significant alterations in whole-body energy homeostasis but exhibit a moderate lipolytic defect, similar as previously observed in MGL−/− mice [24].

**Table 1:** Reduced fasting FG and FFA concentrations in DKO mice

Chow diet						
Feeding state	Fed state (*ad libitum*)		Fasted state (12 h fasting)		Re-fed state (2 h feeding after 12 h fasting)	
Genotype	ApoE−/−	DKO	ApoE−/−	DKO	ApoE−/−	DKO
TG (mg/dl)	56.7 ± 14.9	44.4 ± 9.7	90.2 ± 42.8	64.1 ± 19.5	74.6 ± 11.5	69.9 ± 19.6
TC (mg/dl)	181 ± 43.5	179 ± 48.2	282 ± 40.8	251 ± 41.6	253 ± 40.3	261 ± 33.8
FC (mg/dl)	67.8 ± 14.1	68.5 ± 7.94	81.4 ± 14.8	74.0 ± 13.9	91.0 ± 11.9	91.7± 10.9
CE (mg/dl)	113 ± 30.6	110 ± 43.2	200 ± 27.8	177 ± 28.8	162 ± 32.1	169 ± 26.3
FG (mg/dl)	2.92 ± 0.95	2.83 ± 0.89	3.73 ± 0.74	2.54 ± 0.72**	1.77 ± 0.48	2.75 ± 0.94*
FFA (mmol/l)	0.50 ± 0.18	0.47 ± 0.15	0.92 ± 0.20	0.70 ± 0.23*	0.24 ± 0.03	0.28 ± 0.05

Plasma parameters in chow diet-fed mice aged 6–8 weeks were determined in fed, fasted, and re-fed conditions. Data are represented as mean (*n* = 7–9) ± SD. *p < 0.05; **p ≤ 0.01.

**Table 2:** Reduced fasting FG and FFA concentrations in DKO mice

Western-type diet						
Feeding state	Fed state (*ad libitum*)		Fasted state (12 h fasting)		Re-fed state (2 h feeding after 12 h fasting)	
Genotype	ApoE−/−	DKO	ApoE−/−	DKO	ApoE−/−	DKO
TG (mg/dl)	59.0 ± 22.0	47.3 ± 22.8	53.4 ± 14.9	47.3 ± 16.5	78.6 ± 25.3	106 ± 38.5
TC (mg/dl)	537 ± 80.0	587 ± 153	787 ± 178	646 ± 259	526 ± 63.8	600 ± 113
FC (mg/dl)	210 ± 17.8	235 ± 35.2	264 ± 61.0	236 ± 75.2	183 ± 19.6	211 ± 22.2
CE (mg/dl)	327 ± 67.9	352 ± 122	524 ± 119	411 ± 185	342 ± 52.7	390 ± 111
FG (mg/dl)	2.64 ± 0.48	2.17 ± 0.78	4.75 ± 0.79	3.13 ± 0.46***	2.53 ± 0.37	3.18 ± 0.57*
FFA (mmol/l)	0.61 ± 0.13	0.49 ± 0.23	0.88 ± 0.16	0.70 ± 0.15*	0.36 ± 0.08	0.50 ± 0.11*

Plasma parameters in mice aged 13–15 weeks and fed WTD for 6–8 weeks were determined in fed, fasted, and re-fed conditions. Data are represented as mean (*n* = 7–9) ± SD. *p < 0.05; ***p ≤ 0.001.

### Reduced VLDL secretion and LPL activity in DKO mice

**Figure 3:**
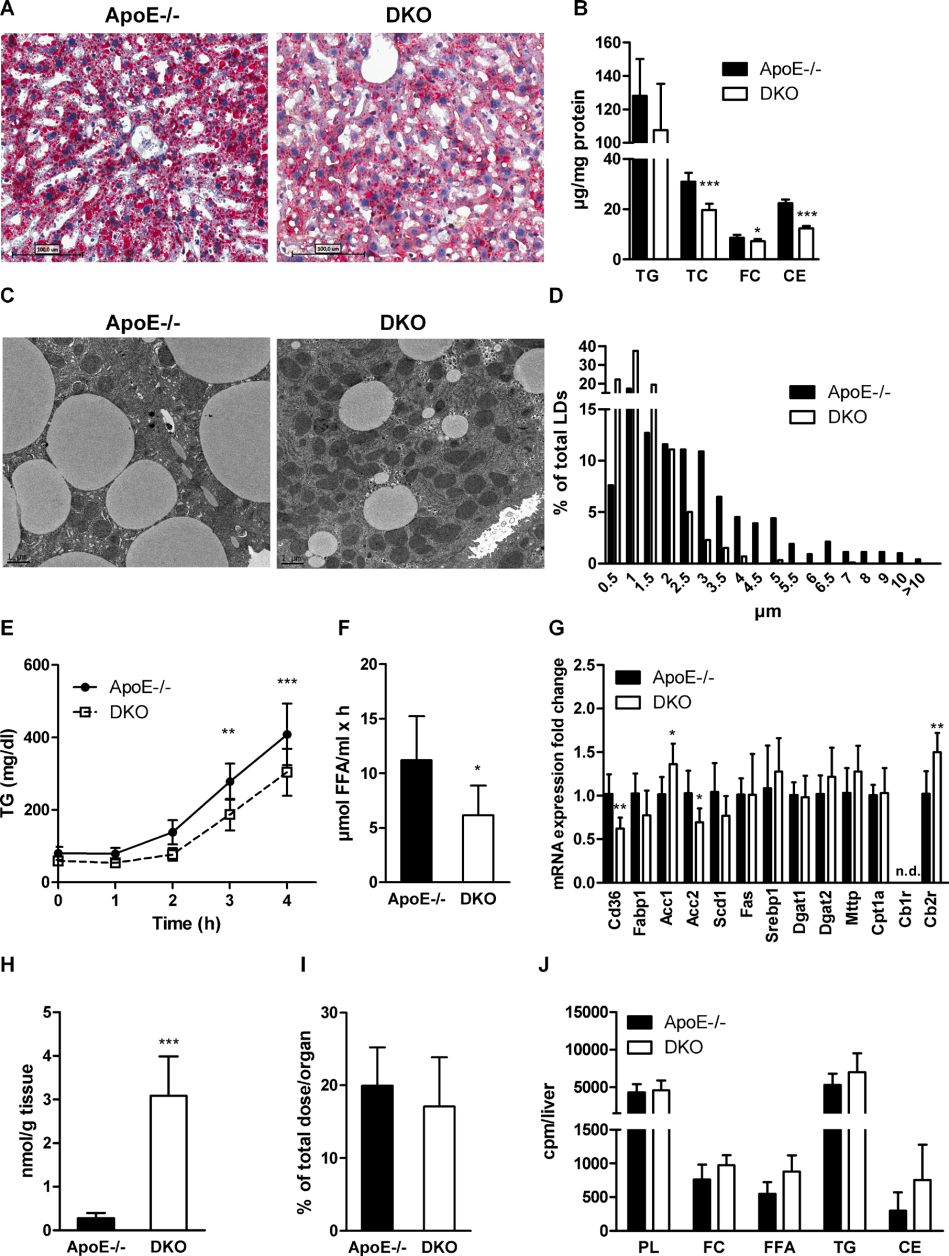
Reduced hepatic steatosis and VLDL secretion in DKO mice (**A**) Oil red O staining (20× magnification), (**B**) biochemical quantification of TG, TC, FC, and CE concentrations (*n* = 6−8), (**C**) electron micrographs, and (**D**) lipid droplet diameters in livers of 12 h-fasted ApoE−/− and DKO mice. (**E**) VLDL secretion of 16 h-fasted mice after tyloxapol injection (*n* = 5–6). (**F**) LPL activity in post-heparinized plasma (*n* = 6−8). (**G**) mRNA expression analyzed in duplicate by real-time PCR and normalized to cyclophilin A expression as reference gene. Expression profiles and associated statistical parameters were determined by the 2*^−ΔΔCt^* method (*n* = 5–7). (**H**) Hepatic 2-AG concentrations measured after 14 weeks of WTD feeding by UPLC-MS (*n* = 7). (I) Hepatic radioactivity content after i.v. injection of [*^3^*H]OA-BSA (*n* = 4–5). (**J**) Hepatic incorporation of i.p. injected [*^14^*C]acetate into lipid classes after TLC separation (*n* = 5–6). Data represent means ± SD; *p < 0.05, **p ≤ 0.01, ***p ≤ 0.001.

Lipolysis is an important regulator of lipid homeostasis during fasting since it provides FFAs as energy source and substrate for hepatic TG synthesis. Oil red O staining of hepatic tissue revealed reduced neutral lipid content in 12 h-fasted DKO mice fed WTD for 16 weeks (Figure 3A). We observed a slight reduction in hepatic TG and significantly decreased TC, FC, and CE concentrations in the livers of DKO mice (Figure 3B). We visualized cytosolic lipid droplets (LDs) by electron microscopy and found reduced LD size in DKO hepatocytes (Figure 3C) with a pronounced shift of the average LD diameter to smaller size (Figure 3D). In fact, 90% of LDs in DKO mice were smaller than 2.5 μm. The total number of LDs was marginally increased (Supplementary Figure 3). Since TG represent the most abundant hepatic lipid pool and serve as a source for VLDL synthesis, we measured the rate of VLDL secretion after an intraperitoneal injection of tyloxapol, which inhibits vascular lipolysis. Three and four hours post-injection, we observed reduced plasma TG concentrations in DKO mice, indicative for decreased VLDL-TG secretion (Figure 3E). Furthermore, we found decreased vascular lipolysis in DKO mice as evidenced by reduced lipoprotein lipase (LPL) activity in post-heparinized plasma (Figure 3F). Additionally, we measured hepatic mRNA expression of genes involved in FA uptake and transport (Cd36, Fabp1), FA synthesis (Acc1, Acc2, Scd1, Fas, Srebp1), TG synthesis and VLDL assembly (Dgat1, Dgat2, Mttp), FA oxidation (Cpt1a), and Cb1r and Cb2r (Figure 3G). Despite 10-fold increased 2-AG concentrations in livers of DKO mice (Figure 3H), transcription levels of the majority of investigated genes were unaltered between the genotypes. We observed only significantly reduced expression of Cd36 and Acc2, whereas Acc1 and Cb2r were upregulated in DKO mice. Cb1r mRNA expression was undetectable in both genotypes. Hepatic uptake of i.v. administered BSA-bound FA, however, was unaltered between genotypes (Figure 3I). Finally, hepatic capacity for lipid synthesis as determined after i.p. injection of [*^14^*C]acetate was unchanged in livers of DKO mice (Figure 3J).

### Reduced hepatic cholesterol content in DKO mice due to increased biliary elimination

**Figure 4:**
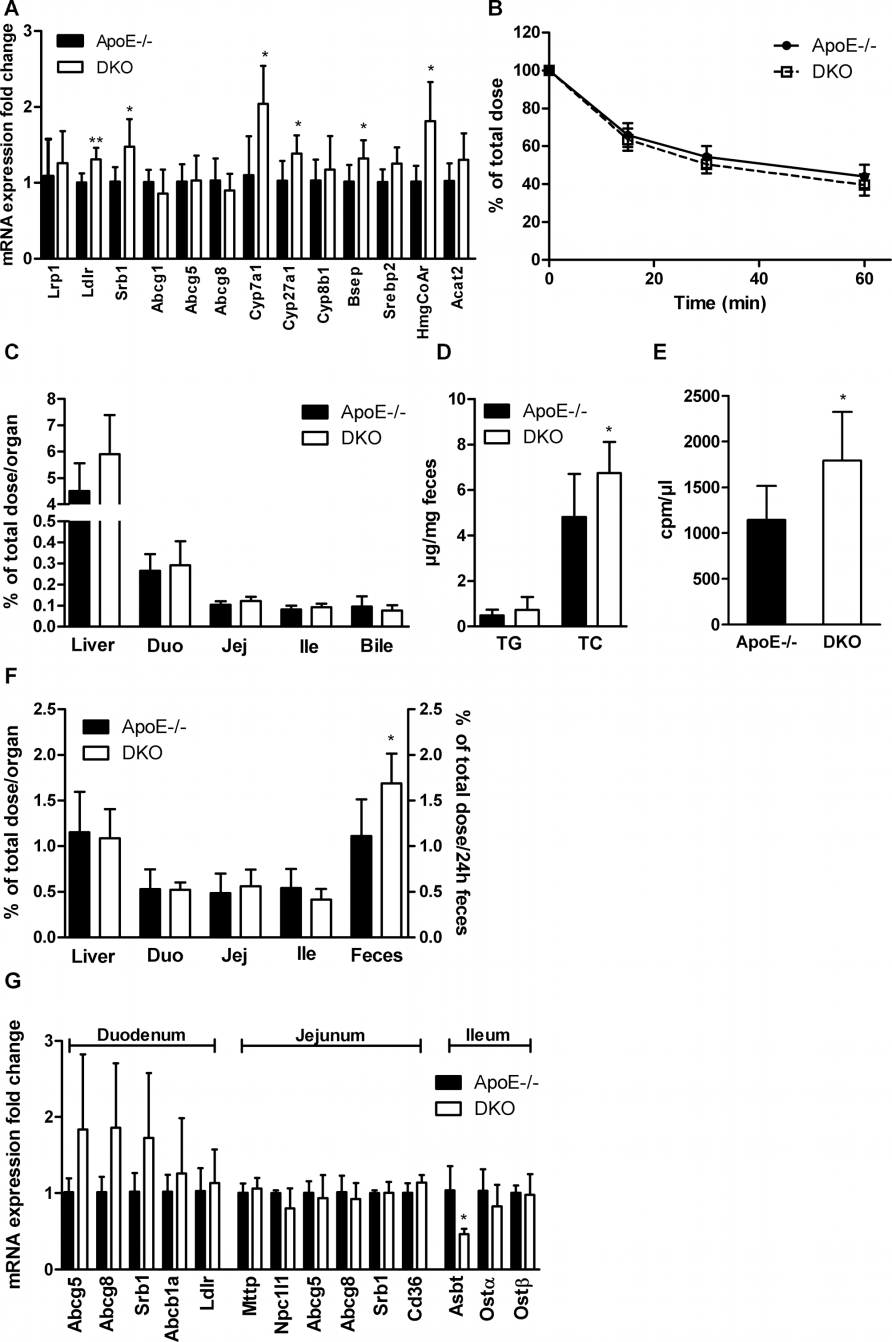
Increased biliary secretion of hepatic cholesterol in DKO mice (**A**) Hepatic mRNA expression analyzed in duplicate by real-time PCR and normalized to cyclophilin A expression as reference gene. Expression profiles and associated statistical parameters were determined by the 2*^−ΔΔCt^* method (*n* = 5–6). (**B**) Plasma and (**C**) tissue radioactivity distribution 60 min after i.v. injection of [*^3^*H]cholesterol-labeled LDL (*n* = 5–6). (**D**) Fecal TG and TC loss (*n* = 8). (**E**) Bile, (**F**) tissue, and fecal radioactivity distribution 24 h after i.v. injection of [*^3^*H]cholesterol-labeled LDL (*n* = 5–6). (**G**) Intestinal mRNA expression analyzed in duplicate by real-time PCR and normalized to cyclophilin A expression as reference gene. Expression profiles and associated statistical parameters were determined by the 2*^−ΔΔCt^* method (*n* = 3−5). Data represent means ± SD; *p < 0.05, **p ≤ 0.01.

Since livers of DKO mice contained significantly reduced amount of cholesterol, we determined mRNA expression of genes involved in lipoprotein and cholesterol uptake (Lrp1, Ldlr, Srb1), cholesterol and bile acid (BA) excretion (Abcg1, Abcg5, Abcg8, Bsep), cholesterol and BA synthesis (Srebp2, HmgCoAr, Cyp7a1, Cyp27a1, Cyp8b1), and cholesterol esterification (Acat2). We found significantly increased expression of Ldlr, Srb1, HmgCoAr, Cyp7a1, Cyp27a1, and Bsep (Figure 4A). To investigate the *in vivo* capacity of DKO livers to take up cholesterol, we injected mice i.v. with [*^3^*H]cholesterol-labeled LDL and chased radioactivity in plasma and tissues. We observed a comparable decrease of radioactivity in plasma (Figure 4B) and similar amounts in liver, all three parts of the small intestine (duodenum, jejunum, ileum), and bile (Figure 4C) in ApoE−/− and DKO mice 60 min after injection. Interestingly, fecal sterol but not TG loss was elevated in the stool of DKO mice fed WTD (Figure 4D). To determine whether cholesterol elimination via the biliary pathway is affected, we injected mice i.v. with [*^3^*H]cholesterol-labeled LDL and measured radioactivity in bile, tissues, and feces 24 h post-injection. While radioactivity in the hepatic and small intestinal tissue was comparable between both genotypes, we found increased radioactivity in the bile (Figure 4E) and feces of DKO mice (Figure 4F), indicating elevated cholesterol/BA excretion and elimination via stool. mRNA expression of genes involved in cholesterol (Abcg5, Abcg8, Srb1, Abcb1a, Ldlr, Npc1l1), FA and TG (Mttp, Cd36), and BA metabolism (Ostα, Ostβ) was unchanged in all parts of the small intestinal tissue. In the ileum of DKO mice, we found reduced mRNA expression of Asbt, which is responsible for ileal BA re-absorption (Figure 4G). Unaltered hepatic cholesterol uptake but increased cholesterol elimination via the biliary pathway indicate that DKO mice exhibit reduced BA re-absorption and, consequently, increased fecal sterol loss.

### Reduced chylomicron secretion and delayed lipid-induced gut transit as well as gastric emptying in DKO mice

**Figure 5:**
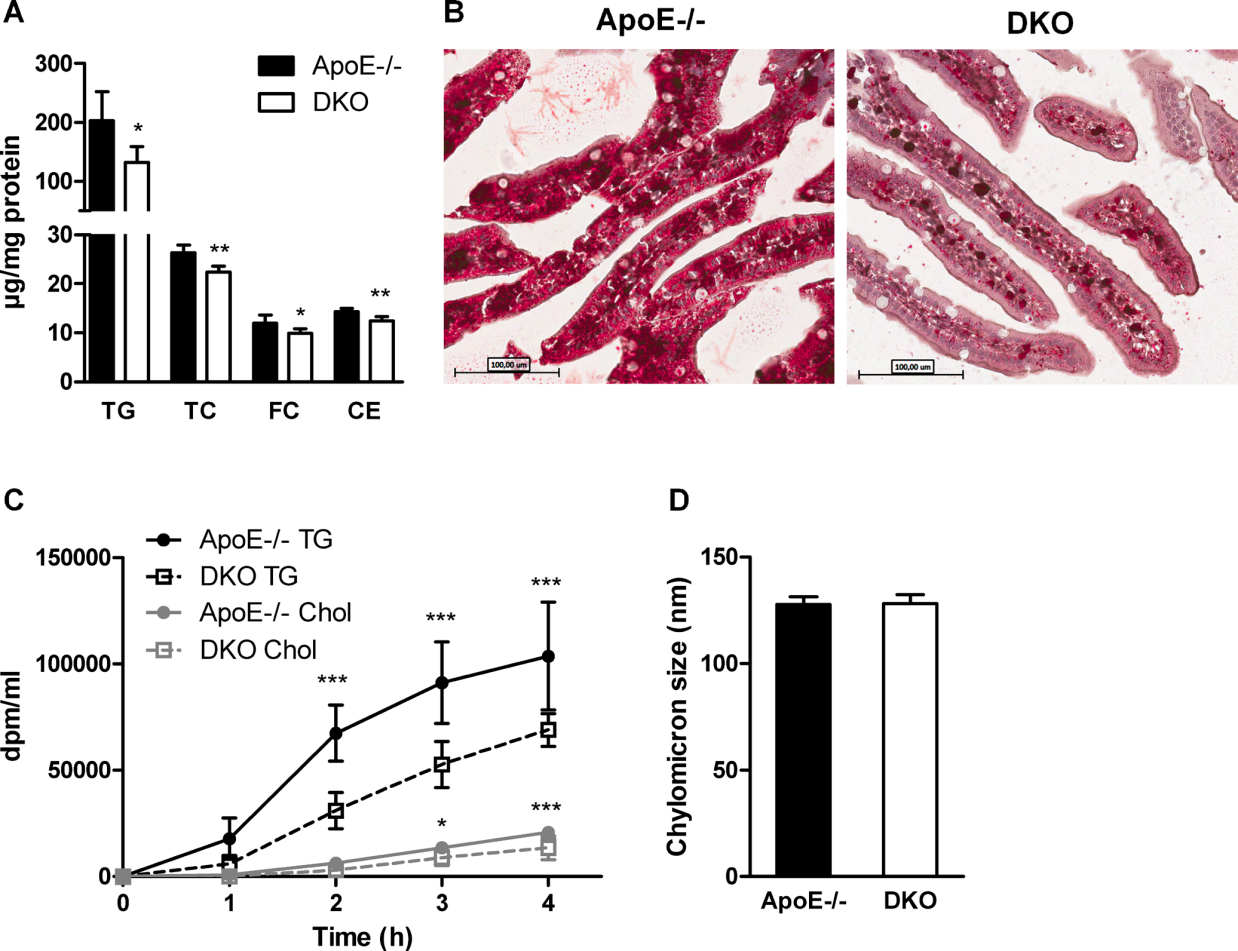
Delayed chylomicron secretion in DKO mice (**A**) Biochemical quantification of (A) TG, TC, FC, and CE in the intestine of WTD-fed and overnight fasted mice 90 min post-gavage with 100 μl corn oil containing 0.25% cholesterol (*n* = 5). (**B**) Representative images of oil red O-stained jejunal sections of the same mice (20× magnification). (**C**) Chylomicron secretion determined as plasma radioactivity distribution after i.p. tyloxapol injection and gavage with corn oil containing [*^3^*H]triolein and [*^14^*C]cholesterol (*n* = 5). (**D**) Chylomicron size measured by light-scattering (*n* = 3). Data represent means ± SD; *p < 0.05, **p ≤ 0.01, ***p ≤ 0.001.

We next investigated whether intestinal lipid metabolism is compromised in DKO mice after administering a lipid bolus. We found significantly reduced concentrations of all investigated lipid classes (TG, TC, FC, CE) in the intestinal tissue of DKO as compared to ApoE−/− mice fed WTD for 16 weeks (Figure 5A). Oil red O staining of jejunal sections confirmed reduced accumulation of neutral lipids in the intestinal mucosa of DKO mice (Figure 5B). To elucidate whether chylomicron secretion is altered in DKO mice, we injected mice i.p. with tyloxapol to inhibit peripheral lipolysis. Thirty min post-injection, we gavaged animals with corn oil containing [*^3^*H]triolein and [*^14^*C]cholesterol. We found significantly reduced appearance of [*^3^*H] and [*^14^*C] in the plasma of DKO mice, indicative for reduced chylomicron secretion (Figure 5C) despite identical chylomicron particle size (Figure 5D).

**Figure 6:**
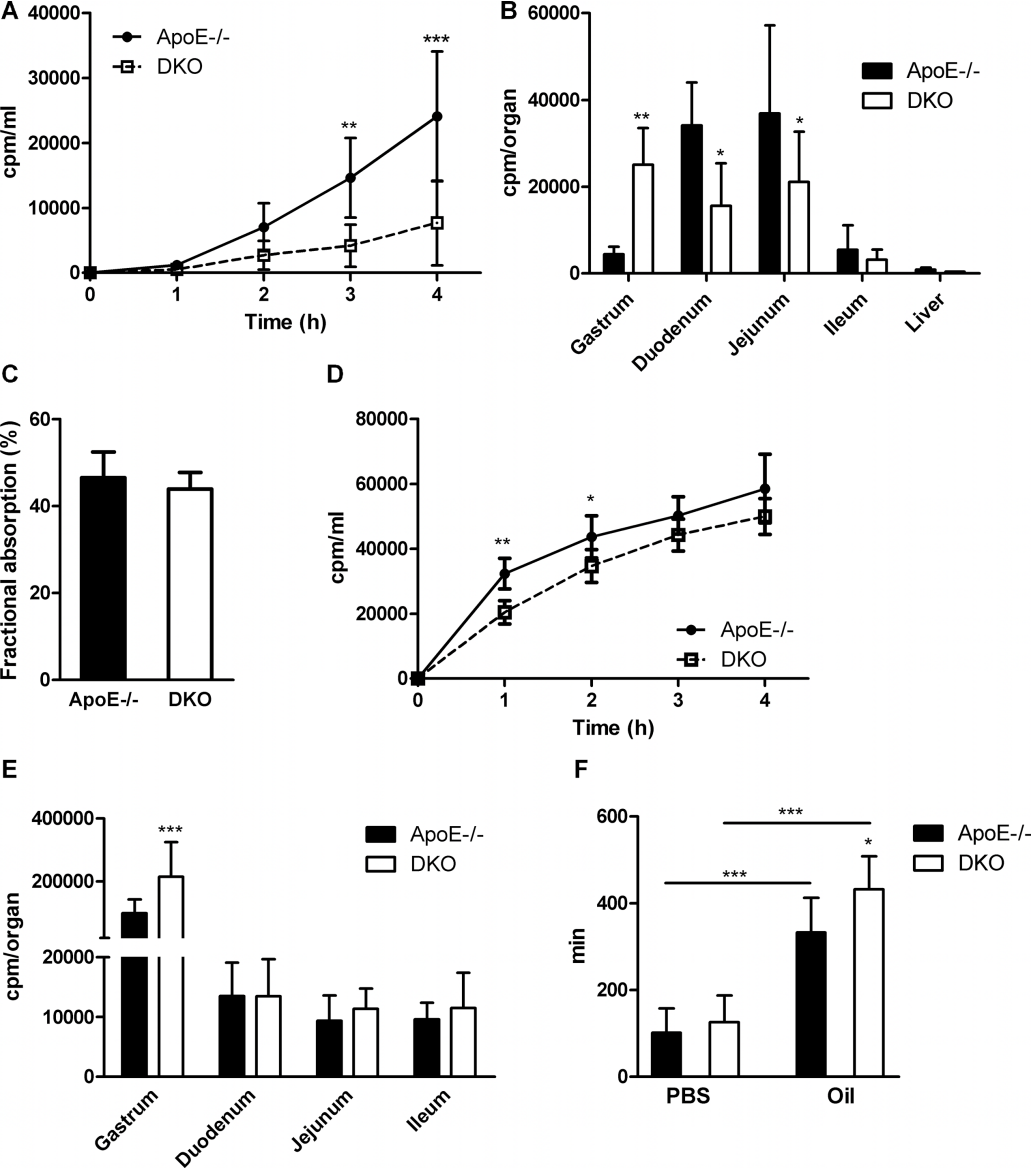
Delayed gastric emptying and reduced gut motility in DKO mice (**A**) Acute cholesterol absorption determined as radioactivity distribution in plasma and (**B**) tissues after corn oil gavage containing [*^3^*H]cholesterol (*n* = 5). (**C**) Fractional cholesterol absorption using the fecal-dual isotope method (*n* = 7). (**D**) Acute FA uptake determined as radioactivity distribution in plasma and (**E**) tissues after corn oil gavage containing [*^3^*H]oleic acid (*n* = 5−6). (**F**) Gut transit time of aqueous- (*n* = 21–22) and olive oil-based suspension (*n* = 9–10) of Evans blue/gum Arabic. Data are presented as means ± SD; *p < 0.05, **p ≤ 0.01, ***p ≤ 0.001.

Reduced accumulation of lipids in intestinal mucosa and their delayed appearance in the circulation may result from reduced lipid uptake. Thus, we measured acute cholesterol absorption and observed reduced distribution of radioactivity in plasma (Figure 6A) and intestinal tissue, but increased retention of the tracer in the gastrum of DKO animals (Figure 6B). Yet, fractional cholesterol absorption was unchanged, indicating that total cholesterol absorption is unaffected (Figure 6C). Similarly, we observed delayed appearance of the radioactive tracer in the plasma (Figure 6D) and increased counts in the stomach, but unchanged radioactivity in the small intestinal tissue of DKO mice (Figure 6E) after gavaging with corn oil containing [*^3^*H]oleate. Finally, to determine whether the observed effects are a consequence of generalized retention of gastric content in DKO mice or due to the chyme composition, we gavaged mice with an aqueous, non-absorptive dye suspension, with or without a preceding oil bolus. We found delayed fecal dye excretion only in DKO mice in combination with an oil bolus (Figure 6F), indicating that the observed effect is dependent on dietary lipid availability. These findings demonstrate that DKO mice do not suffer from intestinal lipid malabsorption but rather show a lipid-induced delay in gastric emptying, which cause a delayed exacerbation of postprandial plasma lipid levels.

## DISCUSSION

In this study, we investigated the physiological consequences of MGL deficiency on lipid and energy homeostasis in an atherosclerotic mouse model. We used ApoE−/− mice to potentiate the effects of aberrant lipid signaling and exacerbate consequences of hyperlipidemia, which affects ApoB-containing lipoprotein particles originating from both intestine and liver. In line with previous findings [24] but in contrast to some other studies in MGL−/− mice [22, 31], DKO mice show unaltered body weight and composition. The controversy in body weights among different studies has been elaborated in detail elsewhere [31]. Moreover, mice on ApoE−/− background suffer from resistance to diet-induced obesity [32], which additionally may hinder a potential difference in body weight of DKO mice compared to control animals. Furthermore, water consumption, locomotor activity, and energy expenditure were unchanged, arguing against hyperactivation of the endocannabinoid system. Yet, we observed distinct changes in lipid metabolism and in the regulation of gut transit that may occur due to aberrant lipid signaling.

Published data suggest that CB1R activation in hepatic tissue causes steatosis due to upregulated lipogenesis [11, 18, 20, 33] and reduced FA oxidation [11, 33], and the opposite occurs by CB1R blockade [33, 34]. Despite increased 2-AG concentrations, MGL−/− mice do not develop hepatic steatosis [24] and are protected from hepatic injury by a mechanism that involves increased endocannabinoid signaling via CB2R and reduced production of eicosanoids in the liver [35]. In fact, we found reduced hepatic TG levels and reduced VLDL secretion in the fasted state in DKO mice. Yet, we failed to observe differences in fasting plasma TG concentrations between ApoE−/− and DKO mice, contrasting reductions in plasma TG levels in MGL−/− mice [24, 31]. It is, however, plausible that changes in plasma TG concentrations are masked in our animal model due to defective VLDL secretion in ApoE−/− animals [36]. Indeed, after prolonged fasting and inhibited peripheral lipolysis, we detected decreased VLDL secretion. Notably, LPL activity was also reduced, which in combination with reduced VLDL synthesis may lead to unaltered plasma TG concentrations in these mice. Thus, the hepatic TG phenotype does not show pro-lipogenic features as would have been expected by CB1R activation. In contrast, it is more likely that hepatic TG synthesis and VLDL secretion are affected by the reduced FA release from peripheral lipolysis during fasting, as reported for MGL−/− mice [24], particularly since hepatic FA uptake and synthesis are not affected by MGL deficiency.

The reduction in hepatic cholesterol concentration as compared to TGs was even more pronounced in DKO mice. Possible reasons include (1) reduced hepatic *de novo* synthesis, (2) reduced uptake of peripheral or alimentary cholesterol, and/or (3) increased biliary/intestinal cholesterol elimination. We failed to observe alterations in either *de novo* synthesis or peripheral LDL-mediated cholesterol uptake despite elevated mRNA expression of Ldlr, Srb1, and HmgCoAr. Increased transcript levels of these genes, however, may be a consequence of a compensatory upregulation due to reduced FC availability within hepatocytes, but with no functional consequence on cholesterol uptake and synthesis. More importantly, the key genes responsible for BA synthesis from cholesterol and secretion into bile were elevated. After injection of [*^3^*H]cholesterol-labeled LDL, we found increased radioactivity in bile and feces of DKO mice, suggesting a modulation of the biliary pathway as the possible mechanism of reduced hepatic cholesterol levels. In addition to increased cholesterol elimination via the biliary pathway, cholesterol excretion via transintestinal cholesterol efflux (TICE) [37] and reduced BA re-absorption (due to reduced Asbt expression in the ileum) may be contributing factors to reduced cholesterol levels in DKO livers. Although it was shown that BA synthesis is increased upon acute CB1R activation by 2-AG [38], all other phenotypic traits of DKO mice and lack of CB1R mRNA expression in DKO livers contradict the possibility of potentiated CB1R signaling. Whether CB2R affects BA metabolism is currently unknown. Taking into account that CB2R expression was increased in DKO livers and receptor activation is functional in DKO mice [26], it may be plausible that CB2R-mediated activation contributes to the potentiated BA metabolism.

We also observed elevated cholesterol content in the feces of DKO mice, which may originate from reduced absorption of dietary cholesterol or increased cholesterol elimination via biliary-dependent and -independent pathways. Thus, we investigated intestinal lipid metabolism, particularly due to the reported delay in alimentary TG absorption in MGL−/− mice, albeit unaltered gut transit [31]. We found reduced lipid accumulation in the small intestine of DKO mice chronically fed WTD and after an oil gavage, and a slower appearance of TG and cholesterol in the circulation, indicative for delayed chylomicron secretion. This was, however, not a consequence of reduced chylomicron size as found in mice with defective TG re-synthesis, ultimately affecting both TG and TC secretion [39]. In line with MGL−/− mice [25], gut transit was unaffected after gavaging an aqueous-based suspension. Our data suggest that the oil bolus delays gastric emptying in DKO mice, which slows down the appearance of chyme in the intestinal lumen and the entry of lipids into the circulation. It is important to note that the lack of an increase in fecal TG and comparable fractional cholesterol absorption (measured after 72 h) demonstrate that lipid absorption is delayed but operative in DKO mice.

In summary, results from this study suggest that MGL deficiency causes complex changes in lipid metabolism including reduced VLDL secretion, increased cholesterol excretion, and delayed gastric emptying. Whether these metabolic effects are caused by CB2R activation needs to be addressed. MGs also activate other signaling pathways (e.g. GPR119, TRPV1) suggesting that CBR-independent effects contribute to the observed phenotype. However, MGL clearly affects lipid metabolism, inflammation [40], and atherosclerosis in DKO mice, placing MGL as a potential target in the treatment of the metabolic syndrome.

## MATERIALS AND METHODS

### Animals and diets

DKO mice were generated and maintained as described elsewhere [26]. Male mice were fed with Western type diet (WTD, 21% fat, 0.2% cholesterol; Ssniff Spezialdiaeten GmbH, Soest, Germany) at the age of 7–8 weeks for a period of 4–16 weeks. Body weight gain during WTD feeding was monitored weekly at 9:00 am. Food and water consumption, locomotor activity, indirect calorimetry and respiratory exchange ratio (RER) were measured in an interval of 15 min during at least 3 consecutive days (PhenoMaster TSE Systems, Bad Homburg, Germany). Experimental protocols were approved by the Austrian Federal Ministry of Science, Research, and Economy, Division of Genetic Engineering and Animal Experiments, Vienna, Austria (BMWF-66.010/0057-II/3b/2011; BMWF-66.010/0159-II/3b/2012; BMWFW-66.010/0076-WF/II/3b/2014; BMWFW-66.010/0065-WF/V/3b/2015).

### Plasma parameters

Plasma lipid parameters were determined as described elsewhere [26]. Plasma free glycerol (FG) and free fatty acid (FFA) concentrations were measured using Free Glycerol Reagent (Sigma-Aldrich, St. Louis, MO) and NEFA-HR kit (Wako Life Sciences, Mountain View, CA), respectively, according to manufacturers’ instructions.

### Plasma lipoprotein profile

Fast protein liquid chromatography was performed as recently described [26].

### Hepatic and intestinal lipid concentrations

Liver and intestinal samples were obtained from 12 h-fasted ApoE−/− and DKO mice fed WTD for 16 weeks. Intestinal lipid concentrations were determined 90 min post-gavaging of 100 μl corn oil containing 0.25% cholesterol. Tissue lipid concentrations were measured according to a modified Folch extraction method as described [41].

### Very low-density lipoprotein (VLDL) secretion

VLDL secretion was measured in 16 h-fasted mice fed WTD for 12 weeks as previously described [42].

### Hepatic FA uptake

Per mouse, 100 μl of 12 mM Na-oleate in ethanol and 4 μCi of [*^3^*H]oleic acid (OA) were evaporated under a stream of N_2_. Lipids were redissolved in 100 μl saline under constant shaking at 37°C for 15 min. This solution was added dropwise to an equal volume of 20% FA-free BSA in saline under constant shaking at 37°C.

Twelve hour-fasted mice were anesthetized with ketamine/xylazine and intravenously injected with 200 μl of the BSA-OA complex. Sixty seconds after injection, blood was taken to determine 100% saturation dose. Fifteen min after injection, mice were sacrificed by heart perfusion with 10 ml of 1 mM EDTA in PBS. Livers were removed, lyophilized overnight, and lysed with 1 ml of 1 M NaOH at 55°C for 4 h. Plasma was isolated by centrifugation at 1,300 × *g* at 4°C for 10 min. Radioactivity in the liver was measured by liquid scintillation counting and normalized to 100% saturation dose.

### Lipoprotein lipase (LPL) activity assay

Mice fed WTD for 12 weeks were injected intraperitoneally (i.p.) with sodium-heparin in PBS (1 IU/kg body weight; Heparin Gilvasan, Gilvasan Pharma, Vienna, Austria). Blood was taken from *v. facialis* and plasma was isolated by centrifugation at 5,200 × *g* for 7 min at 4°C.

For substrate preparation, 0.5 μCi [*^3^*H]triolein (Perkin Elmer, Waltham, MA) and 920 μg triolein (per sample) were dried under a stream of N_2_. Thereafter, 20 μl of 1% Triton X-100, 20 μl of 1 M Tris-HCl (pH 8.6), and 80 μl dH_2_O were added and the mixture was sonicated on ice 3 times for 1 min each. Then, 40 μl heat-inactivated human serum containing ApoC-II as activator and 40 μl of 10% FA-free BSA were added and vortexed.

Two hundred μl of the substrate were incubated with 10 μl of heparinized plasma in the presence or absence of 50 μl of 5 M NaCl (to inhibit LPL activity) in a water bath for 1 h at 37°C under continuous shaking. The reaction was stopped by the addition of 3.25 ml stop solution (n-heptane:chloroform:methanol, 7:9:10) and 1 ml of 0.1 M potassium carbonate (pH 10.5). Tubes were vortexed and then centrifuged at 3,200 × *g* for 15 min at 4°C. One ml of the upper layer was mixed with 6 ml scintillation cocktail and the radioactivity was determined by liquid scintillation counting. LPL activity was calculated according to formula:

LPL activity = total activity (without NaCl) – hepatic lipase activity (with NaCl)

### *De novo* lipid synthesis

Standard chow diet-fed mice were fasted for 4 h, after which the animals were injected i.p. with [*^14^*C]acetic acid (5 μCi in 200 μl PBS; ARC Inc, St. Louis, MO). Mice were sacrificed 1 h post-injection, livers were isolated and lyophilized, and lipids were extracted with 6 ml chloroform:methanol (2:1, v:v). Extracts were evaporated, re-dissolved in chloroform, and separated by thin-layer chromatography (n-hexane:diethylether:acetic acid; 70:30:1, v:v:v). Radioactivity in bands corresponding to phospholipids (PL), free cholesterol (FC), FFA, TG, and cholesteryl esters (CE) was determined by liquid scintillation counting.

### Oil red O staining

Livers were harvested from mice fed WTD for 16 weeks after a fasting period of 12 h. Small intestines were isolated from overnight fasted mice fed WTD for 16 weeks and 90 min post-gavage of 100 μl corn oil containing 0.25% cholesterol. Tissues were fixed with 10% methanol-free neutral-buffered formalin for 24 h, after which samples were transferred to 30% sucrose until processing. One day before sectioning, tissues were transferred into Neg-50™ frozen section medium (Richard-Allan Scientific, Kalamazoo, MI). Sections (5 μm) were cut at -20°C using a cryostat-microtome (HM 560 Cryo-Star; Microm International GmbH, Walldorf, Germany). Oil red O staining was performed as described [26]. Images were taken with ScanScope T3 whole slide scanner (Aperio Technologies, Bristol, UK).

### Electron microscopy and lipid droplet (LD) size measurement

Mice were fed WTD for 16 weeks and fasted for 12 h prior sacrifice. Mice were perfused with PBS/10% methanol-free neutral-buffered formalin and livers were processed for electron microscopy as described [42]. LDs from 94 electron micrographs and total surface of 30,530 μm² per genotype were counted and LD diameter size was analyzed by open source ImageJ software.

### Hepatic 2-AG measurements

Hepatic 2-AG concentrations in mice fed WTD for 12 weeks were measured by UPLC-MS as previously described [26].

### RNA isolation and quantitative real-time PCR analysis

Total RNA from 50 mg tissue was isolated using TriFast™ reagent according to the manufacturer's protocol (Peqlab, Erlangen, Germany). Two μg of total RNA was reverse transcribed using the High Capacity cDNA Reverse Transcription Kit (Applied Biosystems, Carlsbad, CA). Quantitative real-time PCR was performed on a Roche LightCycler 480 (Roche Diagnostics, Palo Alto, CA) using the GoTaq^®^ qPCR MasterMix (Promega, Madison, WI). Samples were analyzed in duplicate and normalized to the expression of cyclophilin A as reference gene. Expression profiles and associated statistical parameters were determined using the 2^−*ΔΔCT*^ method. Primer sequences are listed in the supplemental material.

### Low-density lipoprotein (LDL) uptake

Mice fed WTD for 4 weeks were fasted for 4 h and then injected intravenously (i.v.) with 200 μl of native LDL (2.75 mg/ml cholesterol and 2 μCi [*^3^*H]cholesterol; ARC Inc, St. Louis, MO). Radioactivity in plasma after 1 min of injection was set to 100%. Radioactivity in plasma was chased for 15, 30, and 60 min, after which the animals were sacrificed. Tissues were lyophilized for 24 h, digested in 1 ml of 1 M NaOH, and radioactivity in tissues and plasma was determined by liquid scintillation counting.

### Fecal lipid loss

Mice were fed WTD for 12 weeks and then placed on a fasting grid with unlimited access to food and water. Feces were collected for 72 h, lyophilized, pulverized, and 100 mg were extracted using 8 ml of n-hexane:isopropanol (3:2, v:v). Extracts were separated by centrifugation (3,100 × *g*), decanted, mixed with 200 μl of 2% Triton X-100 in chloroform, evaporated under a stream of nitrogen, and redissolved in 200 μl dH_2_O. Fecal lipid content was measured using enzymatic test kits (Triglycerides FS, Cholesterol FS; DiaSys, Holzheim, Germany).

### Biliary sterol secretion

Mice were fed WTD for 4 weeks, fasted for 6 h, and injected i.v. with 200 μl of native LDL (2.75 mg/ml cholesterol and 2 μCi [*^3^*H]cholesterol; ARC Inc, St. Louis, MO). Animals were placed on fasting grids and had free access to food and water for the next 20 h, after which they were fasted for 4 h and sacrificed. Fecal samples and tissues were lyophilized and lipids were extracted as described above. Bile was isolated by centrifugation at 10,000 × *g* for 10 min from complete gall bladders. Extracts were redissolved in 200 μl of 2% Triton X-100 in dH_2_O and mixed with 1 ml methanol. Tissues were digested in 1 ml of 1 M NaOH and radioactivity in tissues, fecal extracts, and bile was determined by liquid scintillation counting.

### Chylomicron secretion

Chow diet-fed mice were fasted for 4 h and then i.p. injected with 500 mg/kg tyloxapol (Sigma-Aldrich, St. Louis, MO) to inhibit peripheral lipolysis. Thirty min post-injection, mice were gavaged with 200 μl corn oil containing 500 μg cholesterol, 2 μCi [*^3^*H]triolein (Perkin Elmer, Waltham, MA), and 0.5 μCi [*^14^*C]cholesterol (ARC Inc, St. Louis, MO). Radioactivity in plasma was measured 1, 2, 3, and 4 h post-gavage by liquid scintillation counting.

### Chylomicron size measurement

Chow diet-fed mice were i.p. injected with tyloxapol (500 mg/kg; Sigma-Aldrich, St. Louis, MO). Thirty min post-injection, animals were gavaged with 200 μl corn oil. Ninety min post-gavage, plasma was isolated and chylomicrons were separated by density-gradient centrifugation. Particle size was analyzed by dynamic light scattering using a Zetasizer Nano ZS (Malvern Instruments Ltd., Malvern, UK) as described [39].

### Acute cholesterol and FA uptake

Chow diet-fed mice were fasted for 4 h, after which animals were gavaged with 200 μl corn oil containing 2 μCi [*^3^*H]cholesterol (ARC Inc, St. Louis, MO) and 0.25% cholesterol for quantitation of cholesterol uptake, or 100 μl corn oil containing 2 μCi [*^3^*H]OA (Amersham plc, Amersham, UK) to measure FA uptake. Every hour post-gavage, blood was collected and plasma isolated as described above. Four hours post-gavaging, animals were sacrificed and tissues were isolated. Tissues were lyophilized for 24 h, digested in 1 M NaOH, and radioactivity in plasma and tissues was determined by liquid scintillation counting.

### Fractional cholesterol absorption

Mice fed WTD for 4 weeks were fasted for 6 h and then gavaged with 100 μl of corn oil containing 50 μg cholesterol, 0.2 μCi [*^3^*H]sitostanol (ARC Inc, St. Louis, MO), and 0.1 μCi [*^14^*C]cholesterol (ARC Inc, St. Louis, MO). Feces were collected daily for 3 consecutive days, lyophilized, and pulverized. Fecal lipids were extracted from approximately 100 mg of pulverized sample by rotation for 3 h at RT using hexane:isopropanol (3:2, v:v) in 80-fold excess. Extracts were centrifuged at 3,200 × *g* for 15 min at 4°C and the organic phase was decanted to a fresh vial. Two hundred μl of 2% Triton X-100 in chloroform was added to the extracts, vortexed, and dried under a stream of N_2_. Thereafter, samples were redissolved in 200 μl dH_2_O and 1 ml MeOH, and radioactivity in the extracts was determined by liquid scintillation counting. Fractional cholesterol absorption was calculated by the following formula: % absorption = ((dose [*^14^*C]:[*^3^*H] - fecal [*^14^*C]:[*^3^*H]) / dose [*^14^*C]:[*^3^*H])*100.

### Gut transit time

Chow diet-fed mice were fasted for 12 h, gavaged with 100 μl of 5% gum Arabic suspension in PBS containing 5% Evans blue (aqueous-based) or gavaged with 100 μl olive oil 30 min before Evans blue/gum Arabic suspension gavage (oil based). The time until the appearance of the first colored stool sample was recorded.

### Magnetic resonance imaging (MRI) and body composition analysis

Mice (7–8 weeks of age) were fed WTD for 8 weeks. MRI scans of anesthetized mice were acquired by 3T-MRI (Siemens Tim-Trio, Erlangen, Germany) equipped with a circular polarized animal-coil with an inner-diameter of 3.5 cm (RAPID Biomedical GmbH, Rimpar, Germany). To present WAT depots, T1 weighted images were acquired with a 2D Fast-Spin-Echo Sequence with TR = 565 ms, TE = 14 ms, FOV of 69 × 28 mm, matrix = 448 × 182 and 21 sagittal slices with 1 mm thickness and a distance factor of 10%. For quantification of body fat content an IDEAL fat-water separation technique [43] was employed using 4 consecutive 2D-Gradient-Echo acquisitions with different echo times (TE = 4.92/5.54/6.15/6.77 ms), TR = 411 ms, alpha = 44°, FOV of 37 mm, matrix = 128 and 32 axial slices to cover the whole mouse. Body fat content (percentage of protons bound to triglycerides compared to total proton density) was calculated as in [43] using MATLAB (Mathworks Inc., MA).

### Statistics

Statistical analyses were performed using GraphPad Prism 5.1 software. Statistically significant differences were determined by Student's unpaired *t*-test and Welch correction (in case of unequal variances) for two group comparisons. Multiple group comparison was calculated by ANOVA followed by Bonferroni correction. Data represent mean values ± SD. Values of *p* < 0.05 were considered statistically significant.

## SUPPLEMENTARY MATERIALS FIGURES



## Abbreviations

2-AG: 2-arachidonoylglycerol
ApoE: apolipoprotein E
BSA: bovine serum albumin
CBR: cannabinoid receptor
CE: cholesteryl ester
DKO: double knockout
FC: free cholesterol
FFA: free fatty acid
FG: free glycerol
LPL: lipoprotein lipase
MGL: monoglyceride lipase
PL: phospholipid
TG: triglyceride
VLDL: very low-density lipoprotein
WTD: Western type diet

## References

[1] Vaughan M, Berger JE, Steinberg D (1964). Hormone-Sensitive Lipase and Monoglyceride Lipase Activities in Adipose Tissue. J Biol Chem.

[2] Tornqvist H, Belfrage P (1976). Purification and some properties of a monoacylglycerol-hydrolyzing enzyme of rat adipose tissue. J Biol Chem.

[3] Savinainen JR, Jarvinen T, Laine K, Laitinen JT (2001). Despite substantial degradation, 2-arachidonoylglycerol is a potent full efficacy agonist mediating CB1 receptor-dependent G-protein activation in rat cerebellar membranes. Brit J Pharmacol.

[4] Gonsiorek W, Lunn C, Fan X, Narula S, Lundell D, Hipkin RW (2000). Endocannabinoid 2-arachidonyl glycerol is a full agonist through human type 2 cannabinoid receptor: antagonism by anandamide. Mol Pharmacol.

[5] Hansen KB, Rosenkilde MM, Knop FK, Wellner N, Diep TA, Rehfeld JF, Andersen UB, Holst JJ, Hansen HS (2011). 2-Oleoyl glycerol is a GPR119 agonist and signals GLP-1 release in humans. J Clin Endocrinol Metab.

[6] Sugiura T, Kishimoto S, Oka S, Gokoh M (2006). Biochemistry, pharmacology and physiology of 2-arachidonoylglycerol, an endogenous cannabinoid receptor ligand. Prog Lipid Res.

[7] Savinainen JR, Saario SM, Laitinen JT (2012). The serine hydrolases MAGL, ABHD6 and ABHD12 as guardians of 2-arachidonoylglycerol signalling through cannabinoid receptors. Acta Physiol (Oxf).

[8] Walker JM, Huang SM (2002). Cannabinoid analgesia. Pharmacol Ther.

[9] Rodriguez de Fonseca F, Del Arco I, Martin-Calderon JL, Gorriti MA, Navarro M (1998). Role of the endogenous cannabinoid system in the regulation of motor activity. Neurobiol Dis.

[10] Izzo AA, Sharkey KA (2010). Cannabinoids and the gut: new developments and emerging concepts. Pharmacol Ther.

[11] Osei-Hyiaman D, Liu J, Zhou L, Godlewski G, Harvey-White J, Jeong WI, Batkai S, Marsicano G, Lutz B, Buettner C, Kunos G (2008). Hepatic CB1 receptor is required for development of diet-induced steatosis, dyslipidemia, and insulin and leptin resistance in mice. J Clin Invest.

[12] Tedesco L, Valerio A, Dossena M, Cardile A, Ragni M, Pagano C, Pagotto U, Carruba MO, Vettor R, Nisoli E (2010). Cannabinoid receptor stimulation impairs mitochondrial biogenesis in mouse white adipose tissue, muscle, and liver: the role of eNOS, p38 MAPK, and AMPK pathways. Diabetes.

[13] Williams CM, Kirkham TC (2002). Reversal of delta 9-THC hyperphagia by SR141716 and naloxone but not dexfenfluramine. Pharmacol Biochem Behav.

[14] Pacher P, Batkai S, Kunos G (2006). The endocannabinoid system as an emerging target of pharmacotherapy. Pharmacol Rev.

[15] Cota D, Marsicano G, Tschop M, Grubler Y, Flachskamm C, Schubert M, Auer D, Yassouridis A, Thone-Reineke C, Ortmann S, Tomassoni F, Cervino C, Nisoli E (2003). The endogenous cannabinoid system affects energy balance via central orexigenic drive and peripheral lipogenesis. J Clin Invest.

[16] Matias I, Gonthier MP, Orlando P, Martiadis V, De Petrocellis L, Cervino C, Petrosino S, Hoareau L, Festy F, Pasquali R, Roche R, Maj M, Pagotto U (2006). Regulation, function, and dysregulation of endocannabinoids in models of adipose and beta-pancreatic cells and in obesity and hyperglycemia. J Clin Endocrinol Metab.

[17] Pagano C, Pilon C, Calcagno A, Urbanet R, Rossato M, Milan G, Bianchi K, Rizzuto R, Bernante P, Federspil G, Vettor R (2007). The endogenous cannabinoid system stimulates glucose uptake in human fat cells via phosphatidylinositol 3-kinase and calcium-dependent mechanisms. J Clin Endocrinol Metab.

[18] Osei-Hyiaman D, DePetrillo M, Pacher P, Liu J, Radaeva S, Batkai S, Harvey-White J, Mackie K, Offertaler L, Wang L, Kunos G (2005). Endocannabinoid activation at hepatic CB1 receptors stimulates fatty acid synthesis and contributes to diet-induced obesity. J Clin Invest.

[19] Jourdan T, Djaouti L, Demizieux L, Gresti J, Verges B, Degrace P (2010). CB1 antagonism exerts specific molecular effects on visceral and subcutaneous fat and reverses liver steatosis in diet-induced obese mice. Diabetes.

[20] Ruby MA, Nomura DK, Hudak CS, Barber A, Casida JE, Krauss RM (2011). Acute overactive endocannabinoid signaling induces glucose intolerance, hepatic steatosis, and novel cannabinoid receptor 1 responsive genes. PLoS One.

[21] Eckardt K, Sell H, Taube A, Koenen M, Platzbecker B, Cramer A, Horrighs A, Lehtonen M, Tennagels N, Eckel J (2009). Cannabinoid type 1 receptors in human skeletal muscle cells participate in the negative crosstalk between fat and muscle. Diabetologia.

[22] Chanda PK, Gao Y, Mark L, Btesh J, Strassle BW, Lu P, Piesla MJ, Zhang MY, Bingham B, Uveges A, Kowal D, Garbe D, Kouranova EV (2010). Monoacylglycerol lipase activity is a critical modulator of the tone and integrity of the endocannabinoid system. Mol Pharmacol.

[23] Schlosburg JE, Blankman JL, Long JZ, Nomura DK, Pan B, Kinsey SG, Nguyen PT, Ramesh D, Booker L, Burston JJ, Thomas EA, Selley DE, Sim-Selley LJ (2010). Chronic monoacylglycerol lipase blockade causes functional antagonism of the endocannabinoid system. Nat Neurosci.

[24] Taschler U, Radner FP, Heier C, Schreiber R, Schweiger M, Schoiswohl G, Preiss-Landl K, Jaeger D, Reiter B, Koefeler HC, Wojciechowski J, Theussl C, Penninger JM (2011). Monoglyceride lipase deficiency in mice impairs lipolysis and attenuates diet-induced insulin resistance. J Biol Chem.

[25] Taschler U, Eichmann TO, Radner FPW, Grabner GF, Wolinski H, Storr M, Lass A, Schicho R, Zimmermann R (2015). Monoglyceride lipase deficiency causes desensitization of intestinal cannabinoid receptor type 1 and increased colonic -opioid receptor sensitivity. Brit J Pharmacol.

[26] Vujic N, Schlager S, Eichmann TO, Madreiter-Sokolowski CT, Goeritzer M, Rainer S, Schauer S, Rosenberger A, Woelfler A, Doddapattar P, Zimmermann R, Hoefler G, Lass A (2016). Monoglyceride lipase deficiency modulates endocannabinoid signaling and improves plaque stability in ApoE-knockout mice. Atherosclerosis.

[27] Ishibashi S, Herz J, Maeda N, Goldstein JL, Brown MS (1994). The two-receptor model of lipoprotein clearance: tests of the hypothesis in “knockout” mice lacking the low density lipoprotein receptor, apolipoprotein E, or both proteins. Proc Natl Acad Sci USA.

[28] Zhang SH, Reddick RL, Piedrahita JA, Maeda N (1992). Spontaneous hypercholesterolemia and arterial lesions in mice lacking apolipoprotein. E. Science.

[29] Kuipers F, van Ree JM, Hofker MH, Wolters H, In't Veld G, Havinga R, Vonk RJ, Princen HM, Havekes LM (1996). Altered lipid metabolism in apolipoprotein E-deficient mice does not affect cholesterol balance across the liver. Hepatology.

[30] Sehayek E, Shefer S, Nguyen LB, Ono JG, Merkel M, Breslow JL (2000). Apolipoprotein E regulates dietary cholesterol absorption and biliary cholesterol excretion: studies in C57BL/6 apolipoprotein E knockout mice. Proc Natl Acad Sci U S A.

[31] Douglass JD, Zhou YX, Wu A, Zadrogra JA, Gajda AM, Lackey AI, Lang W, Chevalier KM, Sutton SW, Zhang SP, Flores CM, Connelly MA, Storch J (2015). Global deletion of MGL in mice delays lipid absorption and alters energy homeostasis and diet-induced obesity. J Lipid Res.

[32] Hofmann SM, Perez-Tilve D, Greer TM, Coburn BA, Grant E, Basford JE, Tschop MH, Hui DY (2008). Defective lipid delivery modulates glucose tolerance and metabolic response to diet in apolipoprotein E-deficient mice. Diabetes.

[33] Jeong WI, Osei-Hyiaman D, Park O, Liu J, Batkai S, Mukhopadhyay P, Horiguchi N, Harvey-White J, Marsicano G, Lutz B, Gao B, Kunos G (2008). Paracrine activation of hepatic CB1 receptors by stellate cell-derived endocannabinoids mediates alcoholic fatty liver. Cell Metab.

[34] Gary-Bobo M, Elachouri G, Gallas JF, Janiak P, Marini P, Ravinet-Trillou C, Chabbert M, Cruccioli N, Pfersdorff C, Roque C, Arnone M, Croci T, Soubrie P (2007). Rimonabant reduces obesity-associated hepatic steatosis and features of metabolic syndrome in obese Zucker fa/fa rats. Hepatology.

[35] Cao Z, Mulvihill MM, Mukhopadhyay P, Xu H, Erdelyi K, Hao E, Holovac E, Hasko G, Cravatt BF, Nomura DK, Pacher P (2013). Monoacylglycerol lipase controls endocannabinoid and eicosanoid signaling and hepatic injury in mice. Gastroenterology.

[36] Mensenkamp AR, Jong MC, van Goor H, van Luyn MJ, Bloks V, Havinga R, Voshol PJ, Hofker MH, van Dijk KW, Havekes LM, Kuipers F (1999). Apolipoprotein E participates in the regulation of very low density lipoprotein-triglyceride secretion by the liver. J Biol Chem.

[37] Tietge UJ, Groen AK (2013). Role the TICE?: advancing the concept of transintestinal cholesterol excretion. Arterioscler Thromb Vasc Biol.

[38] Chanda D, Kim YH, Li T, Misra J, Kim DK, Kim JR, Kwon J, Jeong WI, Ahn SH, Park TS, Koo SH, Chiang JY, Lee CH (2013). Hepatic cannabinoid receptor type 1 mediates alcohol-induced regulation of bile acid enzyme genes expression via CREBH. PLoS One.

[39] Sachdev V, Leopold C, Bauer R, Patankar JV, Iqbal J, Obrowsky S, Boverhof R, Doktorova M, Scheicher B, Goeritzer M, Kolb D, Turnbull AV, Zimmer A (2016). Novel role of a triglyceride-synthesizing enzyme: DGAT1 at the crossroad between triglyceride and cholesterol metabolism. Biochim Biophys Acta.

[40] Nomura DK, Morrison BE, Blankman JL, Long JZ, Kinsey SG, Marcondes MC, Ward AM, Hahn YK, Lichtman AH, Conti B, Cravatt BF (2011). Endocannabinoid Hydrolysis Generates Brain Prostaglandins That Promote Neuroinflammation. Science.

[41] Obrowsky S, Chandak PG, Patankar JV, Povoden S, Schlager S, Kershaw EE, Bogner-Strauss JG, Hoefler G, Levak-Frank S, Kratky D (2013). Adipose triglyceride lipase is a TG hydrolase of the small intestine and regulates intestinal PPARalpha signaling. J Lipid Res.

[42] Radovic B, Vujic N, Leopold C, Schlager S, Goeritzer M, Patankar JV, Korbelius M, Kolb D, Reindl J, Wegscheider M, Tomin T, Birner-Gruenberger R, Schittmayer M (2016). Lysosomal acid lipase regulates VLDL synthesis and insulin sensitivity in mice. Diabetologia.

[43] Reeder SB, Wen Z, Yu H, Pineda AR, Gold GE, Markl M, Pelc NJ (2004). Multicoil Dixon chemical species separation with an iterative least-squares estimation method. Magn Reson Med.

