# Supplementary material for: Monoglyceride lipase deficiency affects hepatic cholesterol metabolism and lipid-dependent gut transit in ApoE−/− mice
Source: Oncotarget. 2017 Mar 23;8(20):33122–36. doi: 10.18632/oncotarget.16529 (PMC5464855; doi:10.18632/oncotarget.16529)
Supplement: Supplementary file 1 [file oncotarget-08-33122-s001.pdf]

Monoglyceride lipase deficiency affects hepatic cholesterol metabolism and lipid-dependent gut transit in ApoE−/− mice

Supplementary Materials

Primer sequences used for qPCR

Abcg1-fwd: 5′-GACGCTGACTATAAGAGAGACC-3′, Abcg1-rev: 5′-GGAGTTGCTCAGGACCTTCTTG-3′; Abcb1a-fwd: 5′-AATGTTTCGTTATGCAGGTTGGC-3′, Abcb1a-rev: 5′-TGGCTCTTTTATCGGCCTCAC-3′; Abcg5-fwd: 5′-AGAGGGCCTCACATCAACAGA-3′, Abcg5-rev: 5′- CTGACGCTGTAGGACACATGC-3′; Abcg8-fwd: 5′-CTGTGGAATGGGACTGTACTTC-3′, Abcg8-rev: 5′-GTTGGACTGACCACTGTAGGT-3′; Acat2-fwd: 5′-GATGGTCTGACAGATGCCTT-3′, Acat2-rev: 5′-AGCACTGGCACAATCTCTT-3′; Acc1-fwd: 5′-GGACTTGGAGCAGAGAACCTTCG-3′, Acc1-rev: 5′-CAAGCTGGTTGTTGGAGGTGTA-3′; Acc2-fwd: 5′-CGCTCACCAACAGTAAGGTGG-3′, Acc2-rev: 5′-GCTTGGCAGGGAGTTCCTC-3′; Asbt-fwd: 5′-GTACAATGGTGGAGCACAGC-3′, Asbt-rev: 5′-GTGCCTGGATCATTGAACCC-3′; Bsep-fwd: 5′-GAACATGACAAACGGAACAAGC-3′, Bsep-rev: 5′-CCCAGTGATTACCCACAACCTT-3′; Cb1r-fwd: 5′-AAGTCGATCTTAGACGGCCTT-3′, Cb1r-rev: 5′-TCCTAATTTGGATGCCATGTCTC-3′; Cb2r-fwd: 5′-ACGGTGGCTTGGAGTTCAAC-3′, Cb2r-rev: 5′-GCCGGGAGGACAGGATAAT-3′; Cd36-fwd: 5′-GCAGGTCTATCTACGCTGTG-3′, Cd36-rev: 5′-GGTTGTCTGGATTCTGGAGG-3′; Cpt1a-fwd: 5′-CTCCGCCTGAGCCATGAAG-3′, Cpt1a-rev: 5′-CACCAGTGATGATGCCATTCT-3′; Cyclophilin A-fwd: 5′-GAGCTGTTTGCAGACAAAGTTC-3′, Cyclophilin A-rev: 5′-CCCTGGCACATGAATCCTGG-3′; Cyp7a1-fwd: 5′-GGGCATCTCAAGCAAACACCATTC-3′, Cyp7a1-rev: 5′-CGGGACTGATCTAGAGGGGGACAC-3′; Cyp8b1-fwd: 5′-CCTCTGGACAAGGGTTTTGTG-3′, Cyp8b1-rev: 5′-GCACCGTGAAGACATCCCC-3′; Cyp27a1-fwd: 5′-CTTCATCGCACAAGGAGAGC-3′, Cyp27a1-rev: 5′-ATGGCTTCCAAGGCAAGGTG-3′; Dgat1-fwd: 5′-TCCGCCTCTGGGCATTC-3′, Dgat1-rev: 5′-GAATCGGCCCACAATCCA-3′; Dgat2-fwd: 5′-AGTGGCAATGCTATCATCATCGT-3′, Dgat2-rev: 5′-TCTTCTGGACCCATCGGCCCCAGGA-3′; Fabp1-fwd: 5′-ATGAACTTCTCCGGCAAGTACC-3′, Fabp1-rev: 5′-GGTCCTCGGGCAGACCTAT-3′; Fas-fwd: 5′-GAAGCCGAACACCTCTGTGCAGT-3′, Fas-rev: 5′-CTCCTTGCTGCCATCTGTATTG-3′; HmgCoAr-fwd: 5′-CTATTGCACCGACAAGAAGCCT-3′, HmgCoAr-rev: 5′-GCCATCACAGTGCCACATACAA-3′; Ldlr-fwd: 5′-CATGTCTGTCACCTGTCAGTCC-3′, Ldlr-rev: 5′-CTTGTCCAAGCTGATGCACTCC-3′; Lrp1-fwd: 5′-ACTATGGATGCCCCTAAAACTTG-3′, Lrp1-rev: 5′-GCAATCTCTTTCACCGTCACA-3′; Mttp-fwd: 5′-GTCAACAGAGAGGCGAGAAG-3′, Mttp-rev: 5′-CTAGCCAAGCCTCTCTTGAG-3′; Npc1l1-fwd:

5′-TGTCCCCGCCTTATACAATGG-3′,
Npc1l1-rev: 5′-CCTTGGTGATAGACAGGCTACTG-3′;
Ostα-fwd: 5′-CCGCAGCCCAGCTCCTGAG-3′,
Ostα-rev: 5′-TGCAGAGCTACTCCAGATCAGA-3′;
Ostβ-fwd: 5′-GTCCAGGGCCAGAAACATCTC-3′,
Ostβ-rev: 5′-TCCTTCTCAGGAGGAACATGCT-3′;
Scd1-fwd: 5′-TTCTTGCGATACACTCTGGTGC-3′,
Scd1-rev: 5′-CGGGATTGAATGTTCTTGTCGT-3′;
Srb1-fwd: 5′-TTTGGAGTGGTAGTAAAAAGGGC-3′,
Srb1-rev: 5′-TGACATCAGGGACTCAGAGTAG-3′;
Srebp1-fwd: 5′-CCATCGACTACATCCGCTTCTT-3′,
Srebp1-rev: 5′-ACTTCGTAGGGTCAGGTTCTC-3′;
Srebp2-fwd: 5′-AAGTCCTGCAGCCTCAAGTG-3′,
Srebp2-rev: 5′-CTGCCATCTGTCTTCAGCGT-3′.

ApoE-/-

DKO

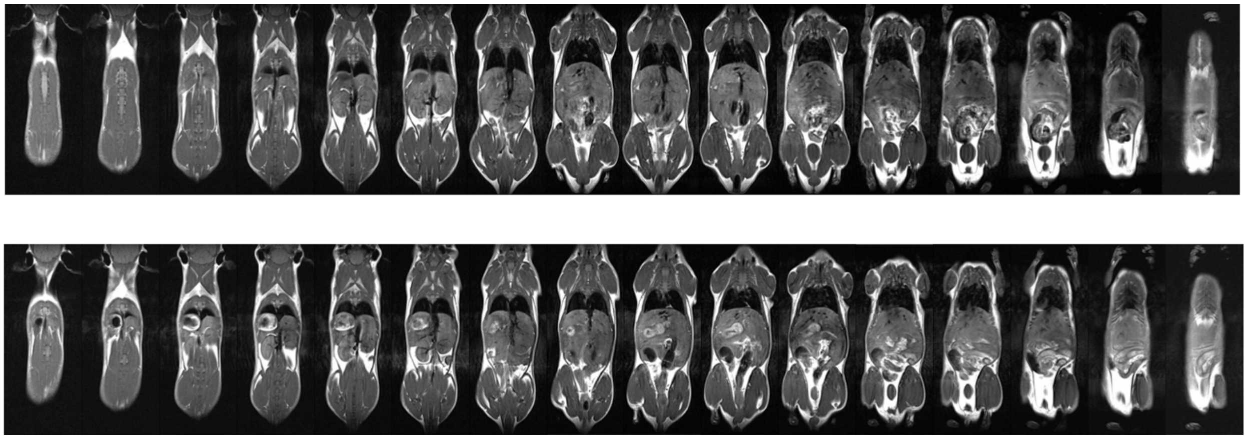

Supplementary Figure 1: Unchanged body fat in DKO mice. Representative images of cross-sectional MRI scan of female ApoE−/− and DKO mice after 9 weeks of WTD feeding. Bright areas represent adipose tissue.

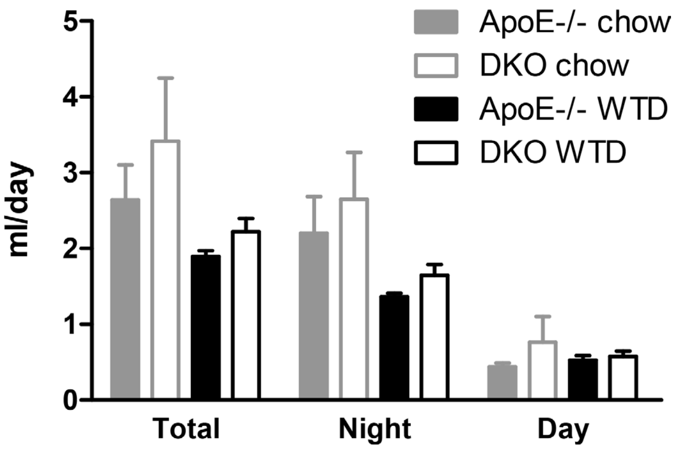

Supplementary Figure 2: Comparable water consumption in ApoE−/− and DKO mice. Water consumption of mice on chow and WTD was monitored by automated sensors in TSE Phenomaster during three consecutive days (*n* = 4–5). Data represent means + SD.

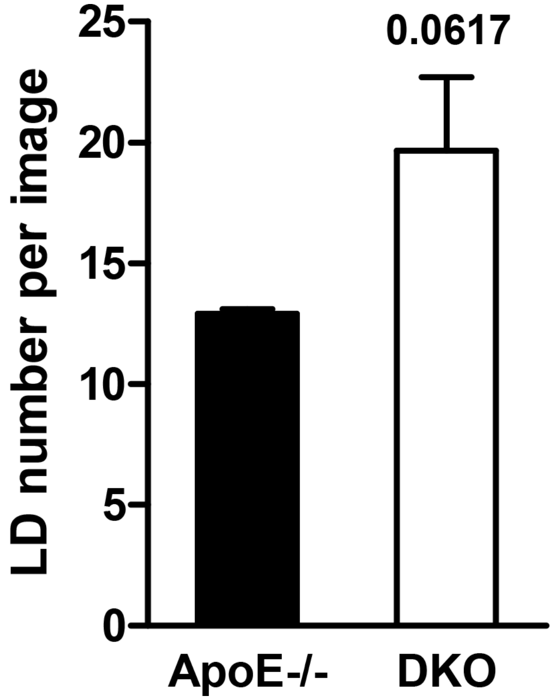

Supplementary Figure 3: Increased LD counts in livers of DKO mice. Mice were fed with WTD and fasted for 16 h before liver isolation. The numbers of LDs per image were counted from 94 electron micrographs and total surface of 30,530 μm² per genotype. Data represent means + SD.
